# New Appliances and Things Medical

**Published:** 1906-05-12

**Authors:** 


### NEW APPLIANCES AND THINGS MEDICAL.

[We shall be glad to receive, at our Office, 28 & 29 Southampton Street, Strand, London, W.C., from the manufacturers, specimens of all new preparations and appliances which may be brought out from time to time.]

### SAJODIN.

(THE BAYER COMPANY, LIMITED, 19 ST. DUNSTAN'S HILL, LONDON, E.C.)

WE have received a sample of the above new iodine preparation. The salt is a colourless, tasteless, insoluble powder, and is sent out in this form; an average dose of the substance is 15 grains. Chemically Sajodin consists of the calcium salt of moniodo-behenic acid and contains 26 per cent. of iodine and 4.1 per cent. of calcium. This substance is intended to replace iodide of potassium, and, indeed, all the iodides at present in therapeutical use; its advantages over these bodies are that it may be given over long periods of time without producing any of the unpleasant effects of the ordinary iodides, which, termed collectively iodism, are so well known to clinicians. There is already a very ample clinical experience published with regard to this drug which is based upon results with 140 patients, all of whom when given sajodin for appropriate diseases showed the therapeutic effect of iodine and did not suffer from iodism. We think this remedy is likely to have a very large sphere of usefulness, and certainly merits an extensive trial.

